# Study on inflammation-related genes and microRNAs, with special emphasis on the vascular repair factor HGF and miR-574-3p, in monocytes and serum of patients with T2D

**DOI:** 10.1186/s13098-015-0113-5

**Published:** 2016-01-15

**Authors:** Lucy Baldeón Rojas, Karin Weigelt, Harm de Wit, Behiye Ozcan, Adri van Oudenaren, Fernando Sempértegui, Eric Sijbrands, Laura Grosse, Anton-Jan van Zonneveld, Hemmo A. Drexhage, Pieter J. M. Leenen

**Affiliations:** Department of Immunology, Erasmus MC, Rotterdam, The Netherlands; Department of Internal Medicine, Erasmus MC, Rotterdam, The Netherlands; Department of Immunology, Central University of Ecuador, Quito, Ecuador; Department of Psychiatry, University of Münster, Münster, Germany; Department of Nephrology, Leiden University Medical Center, Leiden, The Netherlands; Prometeo Program SENESCYT, Central University of Ecuador and Universidad de las Fuerzas Armadas, Quito, Ecuador

**Keywords:** Type 2 diabetes monocytes, Serum, HGF, miR-574-3p

## Abstract

**Background:**

Recently, we reported signs of inflammation (raised IL-8, reduced miR-146a) and signs of vascular repair (raised HGF) in the serum of Ecuadorian patients with type 2 diabetes (T2D). In contrast, we found that the circulating monocytes lacked up-regulation of classical inflammatory genes (IL-1B, IL-6, and TNF) and there was even significant down-regulation of PTGS2. Notably, genes and a microRNA involved in adhesion, cell differentiation and morphology (CD9, DHRS3, PTPN7 and miR-34c-5p) were up-regulated in the T2D monocytes, suggesting a role of the anti-inflammatory cells in adhesion, vascular repair and invasion.

**Aim:**

To determine the gene expression of the vascular repair factor HGF in the circulating monocytes of patients with T2D and to investigate the relationship between HGF and the expression of the other previously tested monocyte genes and the contribution to the raised serum level of HGF. In addition, we tested the level of 6 microRNAs, which were previously found abnormal in the circulating monocytes, in the serum of the patients.

**Methods:**

A gene and microRNA expression study in monocytes and serum of 64 Ecuadorian patients with T2D (37–85 years) and 44 non-diabetic controls (32–87 years).

**Results:**

The gene expression of HGF was significantly raised in the monocytes of the patients with T2D and associated with the expression of genes involved in adhesion, cell differentiation and morphology. HGF gene expression did not correlate with the serum level of HGF. The monocyte expression of pro-inflammatory cytokine genes was also not associated with the serum levels of these cytokines. The level of miR-574-3p was significantly decreased in the serum of the patients with T2D, and correlated in expression with the decreased well-established inflammation-regulating miR-146a. The level of the microRNAs in serum did not correlate with their expression level in monocytes.

**Conclusion:**

In circulating monocytes of Ecuadorian T2D patients, the microRNA and gene expression of important inflammatory/chemotactic/motility/vascular repair factors differs from the expression in serum. While monocytes show a gene expression profile compatible with an anti-inflammatory state, serum shows a molecular profile compatible with an inflammatory state. Both compartments show molecular signs of vascular repair support, i.e. up-regulated HGF levels.

**Electronic supplementary material:**

The online version of this article (doi:10.1186/s13098-015-0113-5) contains supplementary material, which is available to authorized users.

## Background

There is increasing evidence that monocytes, macrophages and related cells are closely involved in the pathogenesis of the metabolic syndrome (MetS) and type 2 diabetes (T2D). Importantly, in obesity the number of macrophages increases from 10–15 % to 50–60 % of total adipose tissue cells [1, 2]. The increase in macrophage number is accompanied by a hyper activation of the cells and leads to a raised secretion of pro-inflammatory cytokines (TNF-α, IL-1β, IL-6, CCL-4, PAI-1) and chemokines (CCL2) causing a state of chronic low-grade inflammation [1, 3–7] and insulin resistance.

Important circulating precursors for the macrophages in the adipose tissue [8–10] are the blood-borne monocytes. There is a relative paucity on the state of inflammatory activation of circulating monocytes in patients with the MetS [11, 12] and T2D [13], but increases in pattern recognition receptors, oxidative stress and the machinery for the production of pro-inflammatory cytokines have all been described [14–16].

Contrary to this view, we recently reported that monocytes of a group of 64 Ecuadorian patients with T2D were characterized by an *anti*-*inflammatory* set point rather than a *pro*-*inflammatory* set point when compared to monocytes of a group of 44 non-diabetic controls. We found a decrease in expression of a cluster of 11 mutually correlated core inflammatory cytokine/compound genes (IL-1B, IL-6, TNF, TNFAIP3, PTGS2, CCL20, CCL2, CCL4, PDE4B, DUSP2 and ATF3; reaching significance for PTGS2) in the monocytes of patients with T2D [17]. However, the study on the monocytes of the patients with T2D also showed that there was up-regulated gene expression for genes occurring in a cluster of mutually correlating genes, many of which are involved in adhesion, migration, cell differentiation and cell morphological change [17]. A significant up-regulation as compared to non-diabetic controls was reached for the genes CD9, DHRS3 and PTPN7. Other important genes in this gene cluster were MAPK6, NAB2, STX1A, EMP-1, CDC42, DHRS3, FABP5, BCL2A1, PTX3 and CXCL2. We interpreted these data as indicating that circulating monocytes in our group of patients with T2D were activated, not towards an inflammatory state, but to a state of enhanced adhesion, migration and further differentiation into descendent cell types, most likely into monocyte-derived pro-angiogenic cells, instrumental in vascular repair. This view was further supported by our observation that the expression of miR-146a, a well-known inflammation down-regulating microRNA, was not changed in the T2D monocytes, while a microRNA targeting genes involved in processes of cell morphology and shape change, i.e. miR-34c-5p, was up-regulated as compared to the group of non-diabetic controls [17].

In the serum of the patients with T2D in whom we performed the monocyte studies, we found clear signs of inflammation [18]. Although there were no increases in the levels of classical cytokines, such as of IL-1β, IL-6 and TNF-α, there was an increase in the level of serum IL-8, and also the level of miR-146a was significantly down-regulated. HGF was increased in the serum of the cases with T2D too [18]. Since HGF is an important vascular repair factor [19–22] and an anti-inflammatory agent [23, 24], and monocyte-derived angiogenic cells are characterized by the expression of HGF [25], we hypothesized that there was an enhanced monocyte-linked endothelial repair mechanism going on in our patients with T2D.

In the present study, we therefore tested the hypothesis that HGF is increased in the circulating anti-inflammatory monocytes of patients with T2D and we determined the gene expression level of HGF (and the HGF-R, cMET) in the monocytes of our patients with T2D and investigated whether HGF belonged to the cluster of typical inflammatory compound genes or to the clusters of typical adhesion, migration and differentiation genes. In addition, we compared the monocyte gene expression levels of HGF to the serum HGF levels to investigate whether the circulating monocytes could be the main producers of this vascular repair factor in serum.

In addition, we measured the serum level of miR-34c-5p, the microRNA up-regulated in the monocytes of patients with T2D and playing a role in cell shape processes, to see whether this microRNA was also raised in the serum of the patients. Finally, we determined the serum level of the other 5 microRNAs (miR-122, miR-138, miR-410, miR-574-3p and miR-92), which we had previously reported as abnormally expressed in the monocytes of patients with T2D in a finding study [17].

## Patients, materials and methods

### Subjects

A total of 64 subjects diagnosed with diabetes type 2, according to the criteria of The Expert Committee on the diagnosis and classification of Diabetes Mellitus [26]. Patients were recruited in four medical centers of Quito, Ecuador (Eugenio Espejo Hospital, Club de Leones Sur, Fundación Oftalmológica del Valle and Fundación de la Psoriasis) from 2009 until 2012. For demographic and clinical details see Table 1. At the same time, 44 healthy controls with similar ethnical and social background, neither suffering from T2D nor other important medical disorders (including acute infection) served as controls. Controls had to be over 30 years of age, considering the age dependency of T2D [27], and preferably of the same gender as the patients.

**Table 1:** Shows sample sizes, distributions of age, gender, comorbidities, HbA1c/hyperglycemia, BMI, hepatic profile, lipid profile, and medication use of the patient and control groups

	HC	T2D	HC vs. T2D
			p value
Group size n	44	64	
Age mean (range)	53 (32–87)	61 (37–85)	0.00**
BMI mean (range) %	28.7 (23–42)	29.5 (22–49)	*0.471*
Normal	18.2 %	16.1 %	
Overweight	45.5 %	40.3 %	
Obese	36.4 %	43.5 %	
Gender			
Female n (%)	31 (70.5 %)	40 (62.5 %)	NA
Male n (%)	13 (29.5 %)	24 (37.5 %)	NA
Glucose state			
Fasting glucose mg/dL, mean (range) %	88 (60.9–180.5)	146 (59–397)	0.00**
Normal	88.6 %	45.3 %	
High	11.4 %	54.7 %	
HbA1C, mean (range) %	5.6 (3.9–6.9)	7.0 (3.2–12.5)	0.00**
Normal	81.8 %	35.7 %	
High	18.25 %	62.5 %	
Lipid profile			
Total cholesterol mg/dL, mean (range) %	237 (131–328)	237 (143–465)	0.99
Normal	31.8 %	37.5 %	
High	68.2 %	62.5 %	
TG mean mg/dL, mean (range) %	194 (85–547)	205 (76–628)	0.56
Normal	63.6 %	60.9 %	
High	36.4 %	39.1 %	
HDL mean mg/dL, mean (range) %	43 (27–87)	43 (17–85)	0.81
Normal	54.5 %	57.8 %	
High	45.5 %	42.2 %	
LDL mg/dL, mean (range) %	158 (78–266)	158 (77–395)	0.95
Normal	50 %	56.3 %	
High	50 %	43.8 %	
Hepatic profile			
ASAT mean mg/dL, mean (range) %	41.3 (19–95)	33.3 (6.0–78)	0.01*
Normal	48.7 %	70.8 %	
High	51.3 %	29.2 %	
ALAT mean mg/dL, mean (range) %	44.7 (10–135)	38.8 (7.0–131)	0.252
Normal	47.4 %	64.6 %	
High	52.6 %	35.4 %	
Medication			
Metformin	0 %	44.6 %	
Insulin	0 %	9.2 %	
Both medications	0 %	15.4 %	
Any anti-diabetic medication	100 %	30.8 %	
Statins (%)	0 %	0 %	

Level of total cholesterol (TC) more than 200 mg/dL, triglycerides (TG) more than 200 mg/dL, high-density cholesterol fraction (HDL) <45 mg/dL in women, <40 mg/dL in men and low-density cholesterol fraction (LDL) more than 130 mg/dL was used for the identification of abnormal values

Values in italics denote a significant difference between two groups

* p 0.01; ** p 0.001

Patients and controls with other immune disorders, other serious medical illnesses, recent infections (last 2 weeks), obvious vascular complications such as diabetic foot and ulcers, fever, pregnancy/postpartum, use of statins and LADA patients (patients positive for GAD-65 Abs) were excluded. The Medical Ethical Review Committee of the Ecuadorian Corporation of Biotechnology Quito, Ecuador and the Ethic Committee of the Central University of Quito approved the study. Written informed consent was obtained of all subjects participating in the study. The Ecuadorian Ministry of Health (MSP) gave the permit to export and process the samples in Erasmus MC, Rotterdam, and The Netherlands.

### Blood collection and preparation

In the morning fasting venous blood was collected. Ten mL were collected in a clotting tube and processed within 4 h. Serum was frozen and stored at minus 80 °C for approximately 24 months before testing. Thirty milliliters were collected in tubes containing sodium-heparin for immune cell preparation. From the heparinized blood, peripheral blood mononuclear cell (PBMC) suspensions were prepared in the afternoon by low-density gradient centrifugation, as previously described in detail [28] within 8 h to avoid activation of the monocytes. PBMCs were frozen in 10 % dimethylsulfoxide and stored in liquid nitrogen. This enabled us to test patient and control serum and immune cells in the same series of experiments later.

### Isolation of monocytes

CD14-positive (CD14^+^) monocytes were isolated from frozen PBMCs by a magnetic cell sorting system (MACS; Miltenyi Biotec, Auburn, CA, USA). The purity of monocytes was >95 % (determined by morphological screening after Trypan Blue staining and flow cytometric analysis). As previously reported; the positive vs. negative selection of immune cells did not influence gene expression profiles [29].

### Real time quantitative PCR (qRT PCR) for monocytes

#### mRNA expression in monocytes via TaqMan array cards

For the previous report we had determined the expression of 24 mRNAs in the monocytes of the T2D cases and the non-diabetic controls. RNA had been isolated from monocytes using RNeasy columns (Qiagen, Hilden, Germany), and both this method and quantitative RT-PCR has been described in detail elsewhere [30]. One microgram of RNA was reverse-transcribed using the High Capacity cDNA kit (Applied Biosystems, Foster City, CA, USA). qPCR was performed using custom TaqMan Arrays, format 48 (Applied Biosystems), according to the manufacturer’s protocol and validated against the single RT-qPCR method. Per fill port, 400 ng of cDNA (converted from total RNA) was loaded. PCR amplification was performed using an Applied Biosystems Prism 7900HT sequence detection system with TaqMan Array block. Thermal cycler conditions were 2 min at 50 °C, 10 min at 94.5 °C, and then 30 s at 97 °C, and 1 min at 59.7 °C for 40 cycles. The expressions of ATF3, BCL2A1, CCL20, CCL2, CCL7, CD9, CDC42, CXCL2, DHRS3, DUSP2, EMP1, FABP5, HSPA1A/HSPA1B, IL-1B, IL-6, MAPK6, NAB2, PDE4B, PTGS2, PTPN7, PTX3, STX1A, TNF, and TNFAIP3 were determined using this card system. The PCR amplification of the housekeeping gene ABL was performed for each sample to allow normalization between the samples. We chose ABL as the housekeeping gene because it has previously been shown that ABL was the most consistently expressed housekeeping gene in hematopoietic cells. The SDS software (ABI) was used to collect the data and the RQ Manager Program (ABI) was used to assign, check, and standardize CT values. The Data Assist software (ABI) was used to normalize the data against ABL expression. For threshold cycles below 40, the corresponding mRNA was considered detected, higher cycle numbers were not included in calculations. Data were expressed as cycle threshold (CT) values corrected to ABL (ΔCT = CT gene X − CT housekeeping gene ABL), and as fold change values determined via the CT method (User Bulletin 2; Applied Biosystems) (ΔΔCt method, formula 2^ΔΔCt^) to linear transform the data and to avoid negative values. To correct for inter-assay variance we set the mean of the studied genes found in the healthy control groups in the same assay for each gene to 1, and the fold change (FC) values of the genes in patient monocytes were expressed relative to this set mean of 1 of the healthy controls for the given values.

#### Individual mRNA qRT–PCR assays for HGF, HGF-R, resistin

For the current report we additionally determined in single assays (not using the card system) the gene expression for HGF, the HGF-R (cMET), and resistin using the same cDNA used in the above described experiments (we measured resistin because resistin had also been found raised in the serum of the T2D cases, though just nor reaching significance, p = 0.07) [18]. TaqMan probes and consensus primers for HGF, HGF-R and resistin were provided by Applied Biosystems. PCR amplification of the housekeeping gene ABL was performed for each sample to allow normalization between the samples. Assays were carried out as described by the manufacturers in 15 µL assays. The fold change values between different groups were determined from the normalized CT values as described above for the outcomes of the card assays.

#### Individual microRNA qRT–PCR assays for monocytes

Total RNA was isolated from purified monocytes using RNeasy columns (Qiagen, Hilden, Germany) as described by the manufacturer’s manual. Purity and integrity of the RNA were assessed on the Agilent 2100 bioanalyzer with the RNA 6000 Nano LabChip^®^ reagent set (Agilent Technologies, Santa Clara, CA, USA) and the RNA was then stored at −80 °C. Subsequently, specific stem-looped reverse transcription primers were used to obtain cDNA for mature microRNAs. The RNA was reverse transcribed using the TaqMan^®^ MicroRNA Reverse Transcription Kit from Applied Biosystems, The Netherlands (ABI). PCR was performed using pre-designed TaqMan^®^ microRNA assays and TaqMan^®^ Universal Master Mix, NoAmpErase^®^UNG, with an ABI 7900 HT real-time PCR machine. RNU44 was used as reference microRNA. The PCR conditions were 2 min at 50 °C, 10 min at 95 °C, followed by 40 cycles of 15 s at 95 °C, and finally 1 min at 60 °C. The Data Assist software (ABI) was used to normalize the data to RNU44. Calculations of FC were as described above.

### Serum cytokines

The levels of TNFα, IL-1β, IL-6, NGF, HGF, PAI, resistin, CCL2 (MCP-1), adiponectin, leptin, IL-8, and MIP1β (CCL4) were measured by flow cytometry (BD LSR II Biosciences, CA, and EE.UU.) using a commercially available multi-analyte cytometric bead array system (Milliplex^®^ Map, USA). This multi-analyte assay panel was specifically designed for T2D studies by Millipore and is a relative economic assay using minimal quantities of serum (25 μL). The data were analyzed using a 5-parameter logistic method for calculating analyte concentrations in samples (MAGPIX^®^ with xPONENT software, Luminex, Austin, USA). Serum analyte levels below the detection limit of the assay (only found with one analyte, i.e. IL-1β in 45 % of the determinations) were considered as 0 pg/ml and included as such in the statistical analysis (if e.g. half maximal values of the detection limit had been used slightly higher values would have been obtained, but significance levels had not changed for IL-1β). Lipid and hepatic profile was performed according to standard lab procedures in Quito-Ecuador (AMCOR laboratory) and validated in Erasmus MC.

### Serum microRNA quantitative real-time PCR (qPCR) in serum

Total RNA was isolated from serum using the same Qiagen miRNeasy kit as used for the monocytes. In order to correct for variations in RNA isolation derived, we spiked-in a non-human (*C. elegans*) synthetic miRNA cel-miR-39 miRNA Mimic (MSY000010) into the sample before nucleic acid isolation. Subsequently, specific stem-looped reverse transcription primers were used to obtain cDNA for mature microRNAs. The RNA was reverse transcribed using the TaqMan^®^ MicroRNA Reverse Transcription Kit from Applied Biosystems, The Netherlands (ABI). PCR was performed using pre-designed TaqMan^®^ microRNA assays and TaqMan^®^ Universal Master Mix, NoAmpErase^®^UNG, with an ABI 7900 HT real-time PCR machine. The PCR conditions were 2 min at 50 °C, 10 min at 95 °C, followed by 40 cycles of 15 s at 95 °C, and finally 1 min at 60 °C. The spiked-in syn-cel-miR-39 goes through the entire RNA isolation process and serves as endogenous control for data normalization. The Data Assist software (ABI) was used to normalize the data to syn-cel-miR-39, further calculations of FC were as described above.

### Data analysis

Statistical analysis was performed using the SPSS (IBM, Inc.) package for Windows. Data were tested for normal distribution using the Kolmogorov–Smirnov test. The Grubbs’ test for outlier detection was applied (http://graphpad.com/support/faqid/1598/). Depending on the distribution pattern and the total number of subjects, parametric (normal distribution, independent t test) or nonparametric group comparison (Mann–Whitney U test) were applied. Correlations were determined by Spearman’s correlation coefficient. Levels of significance were set at p ≤ 0.05 (two tailed). A dendrogram visualizing associations was constructed in SPSS using hierarchical cluster analysis of the serum cytokines, genes and microRNA expression using the between-groups linkage method. Graphs were designed with Illustrator CS6 for Windows.

## Results

### HGF is over expressed in monocytes of patients with T2D

**Fig. 1:**
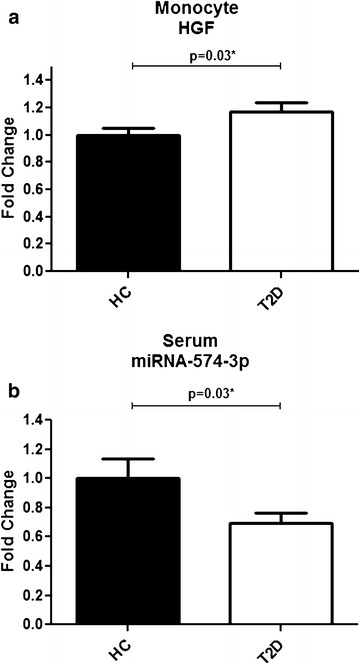
**a, b** Expression level of monocyte HGF and serum miR-574-3p in ecuadorian non-diabetic controls and T2D patients. **a** show mean and standard deviation of the fold change values of HGF (reference gene ABL) in the monocytes of the T2D patients as compared to non-diabetic controls. **b** show mean and standard deviation of the fold change values of miR-574-3p (reference microRNA sync-cel-mir39) in the serum of the T2D patients as compared to non-diabetic controls. Differences between groups were tested using independent T test. Levels of significance were set at p = 0.05 (two tailed)

Table 1 shows the demographic and clinical data of the Ecuadorian type 2 diabetic (T2D) patients and their controls. It is important to note that we were not completely successful in matching the controls to the patients with regard to age. We therefore corrected all further data for age. It is also worthy to note that controls were equally overweight and obese as our patients and had the same abnormal lipid profiles as the patients. Furthermore patients had a better liver profile than the non-diabetic controls. With regard to medication 31 % were not on anti-diabetic medication (recently discovered patients), while 45 % used metformin and 9 % used insulin (15 % were on both medications). None of the patients and non-diabetic controls used statins, Table 2A and Fig. 1a shows that the HGF expression levels were significantly higher in the monocytes of the patients with T2D as compared to the non-diabetic controls [(fold change T2D vs non-diabetic controls) 1.17 ± SEM 0.62, p = 0.03, n = 59]. Controlling for age, gender, dyslipidemia or liver function via hierarchical clustering showed that these factors did not contribute to the association of monocyte HGF expression with disease. With regard to BMI both BMI and T2D were significantly correlated to monocyte HGF expression. There was a non-significant difference in monocyte HGF expression between patients on metformin (FC 1.21 ± 0.37, n = 23) and un-medicated patients (FC 1.09 ± 0.30, n = 8), although group numbers are too small for a valid statistical evaluation.

**Table 2:** Expression level of (A) monocyte genes HGF, HGF-R and resistin and (B) serum microRNAs in non-diabetic controls and patients with T2D

	Non-diabetic controls			T2D			Non-diabetic cont vs. T2D
							P value
	**N**	Mean	SEM	N	Mean	SEM	T test
(A) Monocyte genes^a^							
HGF	27	1.00	0.49	32	1.17	0.62	0.03
HGF-R	12	1.00	0.14	10	1.34	0.36	0.40
Resistin	27	1.00	0.43	32	0.47	0.07	0.24
(B) Serum microRNAs^b^							
miR-122	40	1.00	0.15	56	0.86	0.23	0.64
miR-138	13	1.00	0.24	22	0.87	0.17	0.66
*miR*-*146a*	40	1.00	0.12	56	0.71	0.06	0.02
miR-155	40	1.00	0.07	55	0.95	0.07	0.65
miR-34c-5p	15	1.00	0.11	23	1.00	0.08	0.96
*miR*-*410*	34	1.00	0.12	46	0.92	1.11	0.63
*miR*-*574*-*3p*	37	1.00	0.13	53	0.69	0.07	0.03
miR-576-3p	22	1.00	0.16	36	1.43	0.32	0.31

^a^Group size, mean and SEM of HGF, HGF-R and resistin of monocytes. To avoid inter-assay variation, gene levels were expressed in fold change compared to non-diabetic controls, the average of the controls in each assay was set to one (1.00). Differences between groups were tested using independent T test. Levels of significance were set at p = 0.05 (two-tailed). The HGF expression was significantly higher in the monocytes of the patients with T2D as compared to the non-diabetic controls (p = 0.03)

^b^This table shows group size, mean and SEM of the fold change values of tested serum microRNAs (reference microRNA sync-cel-mir-39) of the patients with T2D as compared to Non-diabetic controls. Differences between groups were tested using independent T test. Levels of significance were set at p = 0.05 (two-tailed). Serum level of miR-146-a (previously reported [18]) and miR-574-3p, was significantly reduced (p = 0.03) in the T2D sera as compared to the non-diabetic controls when controlling for age, gender, BMI and dyslipidemia

The expression levels of the HGF-R (cMET, FC 1.34 ± 0.36 p = 0.40, n = 22) and of resistin (FC 0.47 ± 0.07, p = 0.24, n = 59) were not different between the groups (Table 2A).

**Fig. 2:**
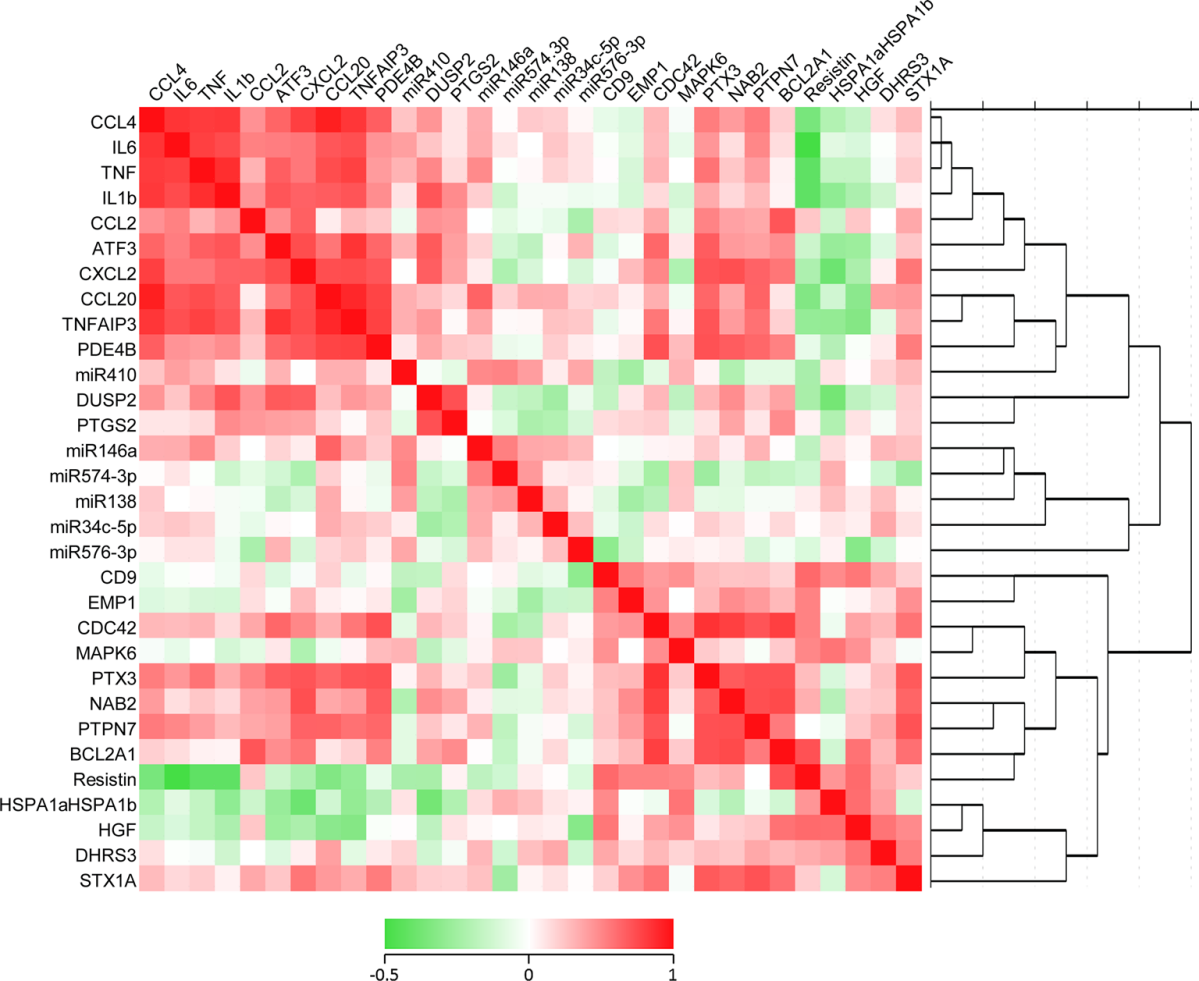
Hierarchical cluster analysis of the tested genes and microRNAs of the monocytes of type 2 diabetic patients and controls. The *figure* show the heat map and cluster diagram for HGF and resistin with the previously determined genes and the previously determined microRNAs. HGF and resistin co-clustered positively with each other and with many genes of the cluster of adhesion/differentiation and shape change genes

Figure 2 shows the heat map and cluster diagram for HGF and resistin with the previously determined genes and the previously determined microRNAs. As can be seen HGF and resistin co-clustered positively with each other and with many genes of the cluster of adhesion/differentiation and shape change genes. Since HGF was significantly over expressed in the T2D monocytes, we focused on this compound. The association of HGF was significant at the p < 0.001 level with DHRS3 (r = .498, p = 0.004, n = 32), CD9 (r = .490, p = 0.004, n = 32), BCL2A1 (r = .503, p = 0.003, n = 32), Resistin (r = .532, p = 0.002, n = 32), HSPA1 (r = .525, p = 0.002, n = 32), but existed also at a lower level for MAPK6 (r = .385, p = 0.03, n = 32) and STX1A (r = .419, p = 0.024, n = 32). It is worthy to note that of these genes HGF, DHRS3 and CD9 were all three significantly higher expressed in the monocytes of cases with T2D as compared to the non-diabetic controls (see [17]).

It is also worthy to note that HGF expression did significantly negatively correlate with the expression of many genes of the inflammatory cluster in the monocytes (such as CCL4, IL-6, TNF, IL1-β, ATF3, CXCL2 and CCL20), reaching significance for TNFAIP3(r = −.350, p = 0.05, n = 32), supporting the concept that HGF is an anti-inflammatory agent. With regard to clinical parameters HGF expression in monocytes did positively correlate with the BMI (r = .327, p = 0.011, n = 59).

As reported previously, HGF was significantly raised in the serum of the patients with T2D as compared to the non-diabetic controls. Interestingly serum HGF correlated with monocyte DHRS3 gene expression (r = .326, p = 0.008, n = 64), but not with monocyte HGF gene expression (r = .189, p = 0.152, n = 59). Neither was there a positive or negative correlation for resistin expression in the monocytes and in the serum (data not shown), nor for any of the pro-inflammatory compounds, such as IL-1B, TNF, IL-6 and CCL2 (data not shown). This makes it unlikely that monocytes are the prime source of HGF, resistin or pro-inflammatory compounds in serum.

### Mir-34c-5p is unaltered, but miR-574-3p is significantly reduced in the serum of patients with T2D

For the previous report on cytokines in T2D serum [18], we had determined the expression of various cytokines, growth factors, miR-146a and miR-155 in the serum of the cases with T2D and the non-diabetic controls. We found IL-8, HGF and resistin (the latter at a significance level of p = 0.07) raised in the serum of the patients with T2D in comparison to the non-diabetic controls, while miR-146a was down-regulated (see also “Background”).

For the current report, we determined the microRNAs miR-34c-5p, miR-122, miR-138, miR-410, miR-574-3p and miR-92 in the serum of the patients with T2D and the non-diabetic controls, since we had also measured these microRNAs in the monocytes of the patients in the previously reported study on gene and microRNA expression in the monocytes. In that study, we reported that miR-34c-5p was significantly up-regulated in the monocytes of the patients with T2D (see also “Background”).

Table 2B shows that in the current study the serum level of miR-34c-5p was not changed in the patients with T2D as compared to the non-diabetic controls. However, the serum level of microRNA miR-574-3p, was significantly reduced in the T2D serum as compared to the non-diabetic controls (see also Fig. 1b). Controlling for age, gender, BMI and dyslipidemia via hierarchical clustering showed that these factors did not contribute to the association of miR-574-3p with disease. With regard to medication there was a trend that particularly in the metformin-medicated group the miR-574 levels were reduced (FC 0.58 ± 0.35, n = 36 to non-diabetic controls), while in the unmediated group FC were only slightly reduced as compared to non-diabetic controls (FC 0.92 ± 0.77, n = 14).

**Fig. 3:**
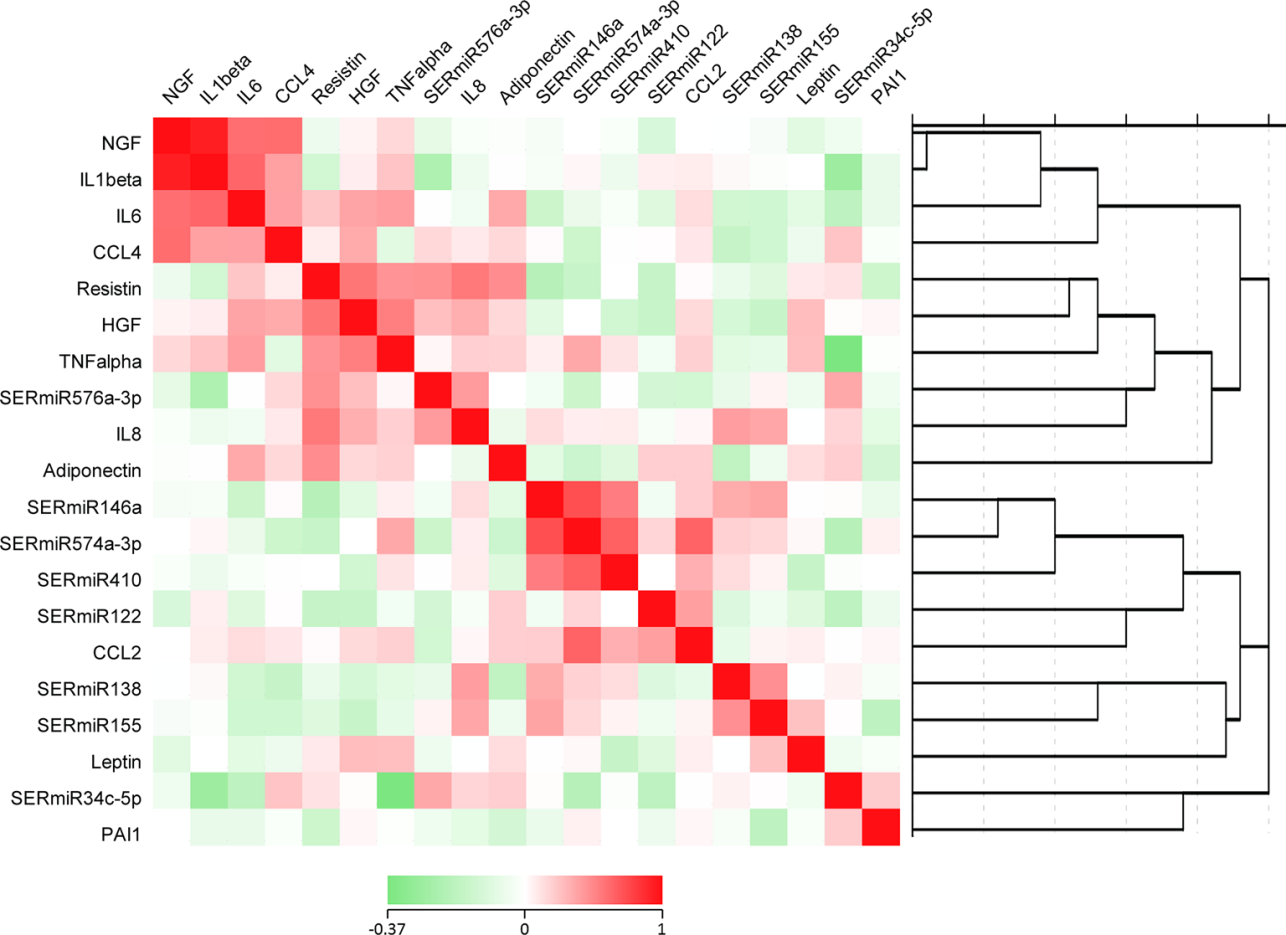
Hierarchical cluster analysis of the tested cytokines and microRNAs of the serum of type 2 diabetic patients and controls. *Figure* shows that there is a strong clustering association of miR-574-3p with miR-146a and miR-410. The association of miR-574-3p with other microRNAs in serum was not strong. The unique association of miR-574-3p with cytokines/growth factors was with the serum CCL2 level

Figure 3 shows the heat map and cluster diagram of the measured microRNAs and cytokines/growth factors. Since miR-574-3p was found significantly reduced in the serum of the patients with T2D, we focused in particular on this microRNA. It is clear from Fig. 3 that there is a strong clustering and association of miR-574-3p with miR-146a (r = 0.744, p < 0.001, n = 88) and miR-410 (r = 0.324, p = 0.03, n = 80), all microRNAs being decreased in the serum of the patients with T2D. The association of miR-574-3p with the other microRNAs and cytokines/growth factors in serum was not very strong, although there was an association with the serum CCL2 level (r = 0.337, p = 0.001, n = 89).

It is also important to note that correlations between the serum levels of the tested microRNAs and the expression levels of the same microRNAs in monocytes were not present (see Table 3).

**Table 3:** Correlations between expression levels of microRNAs tested in monocytes and in serum

Correlation		
Monocyte/serum		
miRNA 138	Correlation coefficient	0.105
	Sig. (2-tailed)	0.560
miRNA 146-a	Correlation coefficient	0.030
	Sig. (2-tailed)	0.788
miRNA 155	Correlation coefficient	0.070
	Sig. (2-tailed)	0.758
miRNA 34c=5p	Correlation coefficient	−0.187
	Sig. (2-tailed)	0.371
miRNA 410	Correlation coefficient	0.108
	Sig. (2-tailed)	0.358
miRNA 574-3p	Correlation coefficient	0.113
	Sig. (2-tailed)	0.305
miRNA 576-3p	Correlation coefficient	−0.066
	Sig. (2-tailed)	0.669

This table shows that correlations between the serum expression levels of the tested microRNAs and the expression levels of the same microRNAs tested in monocytes were not present

### Target prediction for miR-574-3p

Since the expression of miR-574-3p was significantly down-regulated in the serum of patients with T2D, we asked if there were in silico indications linking miR-574-3p expression to processes of inflammation or cell adhesion/differentiation/shape change. We used miRecords as a resource for miRNA-target interactions integrating predicted miRNA targets produced by 11 established miRNA target prediction programs (DIANA-microT, MicroInspector, miRanda, MirTarget2, miTarget, NBmiRTar, PicTar, PITA, RNA22, RNAhybrid and TargetScan/TargetScanS, available at http://www.mirecords.bioled.org).

A minimal target gene prediction coverage of three algorithms was used to perform prediction analysis for miR-574-3p. Filtering to a minimum coverage of three algorithms resulted in 934 hits. Ingenuity pathway analysis (Ingenuity^®^ Systems) was used for mapping of the predicted target genes to biological functions.

Interestingly, the top molecular and cellular function of the miR-574-3p predicted target genes was “cell morphology” and “cellular assembly and organization”, while inflammation did not turn up in any of the predicted pathways (see Additional file 1: Ingenuity analysis).

## Discussion

This study showed that the gene expression of the vascular repair factor HGF was significantly raised in the monocytes of patients with T2D as compared to non-diabetic controls. HGF belonged to the cluster of adhesion, differentiation and shape change genes previously described as up-regulated in the T2D monocytes and correlated significantly to the expression of many genes in that cluster. The association of HGF with these differentiation, adhesion and shape change genes is in accordance with a view that the T2D monocytes are differentiating into the elongated vascular support pro-angiogenic cells (CACs) [25], HGF is a marker of such cells and suggests that the monocytes in our Ecuadorian patients with T2D are instrumental in repairing the vessel walls damaged by T2D related processes.

The up-regulation of HGF in the T2D monocytes might have been instrumental too in the previously reported anti-inflammatory state of the monocytes (see [17]). Indeed there is ample literature on the anti-inflammatory effects of HGF. It has been shown that monocytes treated with HGF produce high levels of IL-10, a potent immune suppressing cytokine. Mechanistically, HGF modulated IL-10 production in monocytes through the ERK1/2 pathway [23]. With regard to dendritic cells (DC), Molnarfi et al. reported that DC differentiated in the presence of HGF adopt a pro-tolerogenic phenotype with increased ability to generate regulatory T cells [24], while with regard to endothelial cells Jeong-Ki Min et al. showed that HGF suppresses vascular endothelial growth factor (VEGF)–induced inflammation by inhibiting the nuclear factor kappa B (NFκB) pathway [30]. In support of this anti-inflammatory action of HGF, we found a negative correlation of intra-monocyte HGF expression with the cluster of inflammatory genes, reaching statistical significance for TNFAIP3 (A20, a molecule induced by TNFα signaling, [31]) expression.

We reported previously that HGF was raised in the serum of the Ecuadorian patients with T2D; however, we did not find a correlation of the serum HGF with the expression of the HGF gene in the monocytes. This suggests that the circulating HGF is not primarily produced by the circulating monocytes, but originates from other sources. This notion also applies to the other tested cytokines and growth factors in the serum of the patients with T2D, for which we could also not find a correlation with intra-monocyte gene expression. Within the serum, the level of HGF correlated with the levels of TNF-α, IL-8 and resistin. Since it is generally thought that these pro-inflammatory compounds and insulin-resistance inducing substances originate from the adipose tissue and the liver [2, 4, 32, 33], we assume that also HGF in the serum of the patients with T2D primarily stems from these sources. There is ample literature on the production of HGF by adipose tissue and the liver [32, 34–37].

Although HGF might originate from sources other than the circulating monocytes, it is nevertheless possible that HGF in the serum could have affected the function of the circulating monocytes in patients with T2D, since the current study shows that monocytes of patients with T2D do express the HGF receptor, though not differently from monocytes of the non-diabetic controls. Interestingly, the level of HGF in serum did not correlate with a reduced inflammatory gene expression in the monocytes, as we found for the intra-monocyte expressed HGF. This suggests that the monocyte-endogenously-produced HGF is more important in the down-regulation of the inflammatory state of the monocytes in the patients with T2D than the serum-borne HGF. The level of circulating HGF did correlate positively to the DHRS3 expression in the monocytes, suggesting that serum-borne HGF might influence the proliferation and differentiation potential of circulating monocytes to pro-angiogenic cells in patients with T2D.

Furthermore, we found that the level of miR-574-3p was significantly reduced in the serum of patients with T2D, similar to miR-146a, of which we reported a down-regulation in the serum of the patients with T2D at an earlier occasion [18]. In the cluster analysis and in correlation studies there was a strong association between the serum level of miR-574-3p and miR-146a, miR-410 and miR-155. This suggests an association of serum miR-574-3p (and also serum miR-410) with inflammatory processes, since miR-146a and miR-155 are important inflammation-regulating microRNAs [38–41]. This notion is further supported by a positive correlation between the serum level of miR-574-3p and the level of CCL2 in serum.

However, when we studied in silico the putative targets of miR-574-3p, ingenuity analysis of the putative targets did not indicate inflammation as an important pathway, whereas cell morphology and cellular assembly and organization were clearly present. The literature on miR-574-3p is in accord with this notion and shows functions of miR-574-3p mainly in the regulation of tumor cell pathology: MiR-574-3p is anti-proliferative, anti-invasive and anti-migratory in gastric and prostate cancer cells [42–44]. In these studies it was found that cullin-1 might be a target of miR-574-3p, and interestingly cullin-1 regulates inflammation via NFκB, thus giving an opening to a relationship with inflammation in connection with cellular assembly and organization [44].

Our findings suggest a high pro-angiogenic potential of the circulating monocytes and the serum of Ecuadorian patients with T2D. It is important to note that Yang reported that down-regulation of miR-574-3p in pro-angiogenic cells appeared to be a marker of senescence; senescent pro-angiogenic cells have lost their proliferative capacity and are changed in an inflammatory (oxidative radicals) direction [45]. Hence, this might indicate that miR-574-3p is involved in the regulation of the anti-inflammatory and pro-angiogenic state of the circulating monocytes of patients with T2D.

Perhaps the most striking observation in the present study is the absence of correlation between the serum and intracellular monocyte levels of cytokines, growth factors and microRNAs. This suggests that the dynamics of the inflammation-related changes in the monocyte intracellular compartment differ substantially from the dynamics of inflammation-related changes in the serum compartment of patients with T2D. With regard to microRNAs it is possible that these dynamics involve complex processes of micro-vesicle or apoptotic body release from immune, endothelial or other cells [46] and/or binding to serum lipoproteins [47], illustrating the complexity of the biological activities of microRNAs in vascular and metabolic disease.

Collectively the data of the previous studies and the current study show that both the monocyte intracellular compartment and the serum compartment of our patients with T2D have undergone inflammation-related changes. However, the monocyte compartment shows in general a reduction in gene expression of typical pro-inflammatory genes, while genes and microRNAs involved in cell adhesion, cell differentiation, growth and vascular repair, such as HGF and miR-34c-5p, are up-regulated. The serum compartment, in contrast to the monocyte compartment, does show signs of high pro-inflammatory activity, e.g. high levels of IL-8 and reduced levels of anti-inflammatory miR-146a and altered levels of miR-574-3p. The serum compartment also shows signs of higher activity of vascular repair and cellular growth induction, yet parameters do not correlate with the monocyte parameters of higher pro-angiogenic cell activity. Most likely different T2D related pathophysiological forces drive the activation and de-activation set points of the circulating monocyte and the serum compartment.

## Limitations

Due to the paucity of material we have not been able to carry out all assays in all patients and thus for some microRNAs (such as e.g. 34c-5p and 576-3p) test numbers might have been too small to detect significant changes. The likelihood that we would have found a significant difference for these microRNAs if we had been able to increase the sample size is possible. However when numbers for miR-574-3p, which we found statistically significant between the groups with n numbers of 53 patients and 37 controls, were recalculated with reduced numbers equal to those for the miRs 34c-5p and 576-3p (with n numbers around half of those for 574-3p), near significant p levels between 0.05 and 0.10 were found. With none of the here tested microRNAs such near significant p levels were found. Nevertheless it is clear that in future studies larger samples need to be tested to come to more solid conclusions.

Although we selected patients and controls on the basis of their diabetic state (glucose levels) to study diabetes as the major determinant for immune differences, we realize that other determinants might have played equally important roles in the outcomes of our study, such as lipid state, liver function, BMI and medication. Correction for lipid state, BMI and liver function did not change our main findings regarding the higher expression of monocyte HGF and reduced serum miR-574-3p. With regard to metformin treatment, which has been shown to alter the immune state [48], post hoc analyses of our data found non-significant differences between metformin treated and not treated patients, in particular that the reduced serum miR-574-3p values were more clear in the metformin treated patients. However numbers in the test groups were too small for a valid statistical evaluation.

Although focus was on HGF in the here reported studies to evaluate the vessel repair quality of the circulating monocytes, other important analytes could have been studied as well, such as adiponectin and leptin. Levels of these adipokines are important in determining atherosclerosis [49] and should therefore be studied in future investigations on this subject. This is the more relevant since we found in preliminary unpublished studies that the adiponectin and leptin serum levels were different between Ecuadorian and Dutch T2D patients. Similar observations on an altered leptin/adiponectin ratio have been done by Bribiescas et al. in studying Ache Amerindians versus US individuals [50].

## Conclusion

Despite the limitations of the here described study we conclude that in circulating monocytes of T2D Ecuadorian patients, the microRNA and gene expression of important inflammatory factors, chemotactic/motility factors and a vascular repair factor differs from the expression in serum. While monocytes show a gene expression profile compatible with an anti-inflammatory state, the serum shows a molecular profile of an inflammatory state, suggesting an intricate feedback network. Both compartments show molecular signs of vascular repair support, i.e. up-regulated HGF levels.

## Additional file

10.1186/s13098-015-0113-5 Ingenuity pathway analysis (Ingenuity® Systems) was used to map the major pathways and processes in which miR-574-3p is involved. The top molecular and cellular function of the miRNA predicted target genes was “cell morphology”; while the second top-associated network was “cell morphology” and “cellular assembly and organization”. We used miRecords as a resource for microRNA-target interactions.

## Abbreviations

Abs: antibodies
A20: tumor necrosis factor a-induced protein (TNFAIP) 3
ABL1: Abelson murine leukemia viral oncogene homolog 1
ATF3: cyclic AMP-dependent transcription factor ATF-3
BCL2A1: Bcl-2-related protein A1
CACs: circulating pro-angiogenic cells
CCL2: chemokine (C–C motif) ligand 2. Monocyte chemotactic protein 1 (MCP1)
CCL20: chemokine (C–C motif) ligand 20
CCL4: chemokine (C–C motif) ligand 4
CCL7: chemokine (C–C motif) ligand 7
CD9: cell division 9
CDC42: cell division control protein 42 homolog
cDNA: complementary Deoxyribonucleic acid
cMET: hepatocyte growth factor receptor
Ct: cycle threshold
CXCL2: chemokine (C–X–C motif) ligand 2
DC: dendritic cell
DHRS3: short-chain dehydrogenase/reductase 3
DUSP2: dual specificity protein phosphatase 2
EMP1: epithelial membrane protein 1
FABP5: fatty acid-binding protein, epidermal
GAD-65: glutamic acid decarboxylase 65
HGF: hepatocyte growth factor
HGF-R: hepatocyte growth factor receptor
HSPA1A: heat shock 70 kDa protein 1A
HSPA1B: heat shock 70 kDa protein 1B
IL-10: interleukin 10
IL-6: interleukin 6
ILB: interleukin 1 beta
LADA: latent autoimmune diabetes in adults
MAPK6: mitogen-activated protein kinase 6
MCP-1: monocyte chemotactic protein 1 (CCL2)
MIP1β: macrophage inflammatory protein 1 beta (CCL4)
miR-122: microRNA-122
miR-138: microRNA-138
miR-146a: microRNA-146a
miR-410: microRNA-410
miR-574-3p: microRNA-574-3p
miR-92: microRNA-92
mRNA: messenger ribonucleic acid
NAB2: NGFI-A-binding protein 2
NFκB: nuclear factor kappa-light-chain-enhancer of activated B cells
PBMC: peripheral blood mononuclear cells
PCR: polymerase chain reaction
PDE4B: cAMP-specific 3′,5′-cyclic phosphodiesterase 4B
PGS2: prostaglandin synthase-2
PTGS2: prostaglandin-endoperoxide synthase 2
PTPN7: protein tyrosine phosphatase non-receptor type 7
PTX3: pentraxin-related protein 3
qPCR: quantitative polymerase chain reaction
RNA: ribonucleic acid
RT-PCR: real-time polymerase chain reaction
STX1A: syntaxin-1A
TNF: tumor necrosis factors
TNFAIP3: tumor necrosis factor, alpha-induced protein 3
