# Supplementary material for: Study on inflammation-related genes and microRNAs, with special emphasis on the vascular repair factor HGF and miR-574-3p, in monocytes and serum of patients with T2D
Source: Diabetol Metab Syndr. 2016 Jan 15;8:6. doi: 10.1186/s13098-015-0113-5 (PMC4714426; doi:10.1186/s13098-015-0113-5)
Supplement: Supplementary file 1 — 10.1186/s13098-015-0113-5 Ingenuity pathway analysis (Ingenuity® Systems) was used to map the major pathways and processes in which miR-574-3p is involved. The top molecular and cellular function of the miRNA predicted target genes was “cell morphology”; while the second top-associated network was “cell morphology” and “cellular assembly and organization”. We used miRecords as a resource for microRNA-target interactions. [file 13098_2015_113_MOESM1_ESM.pdf]

INGENUITY
PATHWAY ANALYSIS

Analysis Name: miRecords_target prediction miR-574-3p_Ingenuity - 2014-08-20 01:50 PM
Analysis Creation Date: 2014-08-20
Build version: 313398M
Content version: 18841524 (Release Date: 2014-06-24)

Analysis settings

View

Reference set: Ingenuity Knowledge Base (Genes Only)

Relationship to include: Direct and Indirect

Includes Endogenous Chemicals

Optional Analyses: My Pathways My List

Filter Summary:

Consider only molecules and/or relationships where

(species = Human) AND

(confidence = Experimentally Observed)

Cutoff:

(c) 2000-2014 QIAGEN. All rights reserved.

Top Canonical Pathways

Name	p-value	Ratio
Huntington's Disease Signaling	9,98E-06	24/226 (0,106)
Molecular Mechanisms of Cancer	1,46E-05	32/359 (0,089)
Glioma Signaling	1,74E-05	14/94 (0,149)
Role of NFAT in Cardiac Hypertrophy	6,58E-05	19/176 (0,108)
GNRH Signaling	1,39E-04	15/127 (0,118)

Top Upstream Regulators

Upstream Regulator	p-value of overlap	Predicted Activation State
mir-145	7,98E-05	
miR-22-3p (miRNAs w/seed AGCUGCC)	3,21E-04	
Vegf	4,98E-04	
CDKN2C	5,15E-04	
AKT2	8,77E-04	

(c) 2000-2014 QIAGEN. All rights reserved.

Top Diseases and Bio Functions

Diseases and Disorders

Name	p-value	# Molecules
Neurological Disease	2,65E-06 - 8,85E-03	143
Developmental Disorder	1,59E-05 - 9,99E-03	84
Hereditary Disorder	5,19E-05 - 9,99E-03	97
Psychological Disorders	5,19E-05 - 8,78E-03	94
Skeletal and Muscular Disorders	5,19E-05 - 9,70E-03	120

Molecular and Cellular Functions

Name	p-value	# Molecules
Cell Morphology	3,49E-09 - 8,80E-03	205
Cellular Assembly and Organization	3,49E-09 - 9,83E-03	173
Cellular Function and Maintenance	3,49E-09 - 9,83E-03	192
Cellular Development	4,56E-09 - 9,83E-03	242
Cellular Growth and Proliferation	2,52E-06 - 9,83E-03	239

(c) 2000-2014 QIAGEN. All rights reserved.

Physiological System Development and Function

Name	p-value	# Molecules
Nervous System Development and Function	1,45E-08 - 9,83E-03	149
Tissue Development	1,45E-08 - 9,83E-03	200
Embryonic Development	1,40E-06 - 9,72E-03	165
Cardiovascular System Development and Function	1,41E-05 - 1,01E-02	92
Organismal Survival	2,53E-05 - 2,75E-03	150

(c) 2000-2014 QIAGEN. All rights reserved.

Top Tox Functions

Assays: Clinical Chemistry and Hematology

Name	p-value	# Molecules
Decreased Levels of Albumin	7,70E-02 - 1,48E-01	1
Increased Levels of CRP	7,70E-02 - 7,70E-02	1
Increased Levels of Potassium	1,29E-01 - 1,29E-01	2
Increased Levels of Red Blood Cells	1,48E-01 - 3,18E-01	5
Increased Levels of ALT	2,14E-01 - 5,33E-01	1

Cardiotoxicity

Name	p-value	# Molecules
Cardiac Proliferation	2,53E-04 - 5,63E-02	11
Cardiac Hypertrophy	1,84E-03 - 8,61E-02	27
Cardiac Arteriopathy	4,51E-03 - 4,06E-01	18
Cardiac Dysfunction	8,39E-03 - 3,03E-01	9
Cardiac Necrosis/Cell Death	8,53E-03 - 3,46E-01	19

(c) 2000-2014 QIAGEN. All rights reserved.

INGENUITY® SYSTEMS

Hepatotoxicity

Name	p-value	# Molecules
Liver Dysplasia	1,11E-03 - 7,70E-02	3
Liver Proliferation	6,23E-03 - 4,30E-01	16
Liver Regeneration	8,78E-03 - 1,13E-01	7
Glutathione Depletion In Liver	1,59E-02 - 4,52E-01	4
Liver Fibrosis	3,93E-02 - 2,60E-01	9

Nephrotoxicity

Name	p-value	# Molecules
Renal Necrosis/Cell Death	1,99E-03 - 4,16E-01	31
Renal Proliferation	2,18E-02 - 1,48E-01	16
Nephrosis	2,78E-02 - 4,30E-01	6
Renal Degradation	3,93E-02 - 3,93E-02	1
Renal Inflammation	7,70E-02 - 4,30E-01	5

Top Regulator Effect Networks

(c) 2000-2014 QIAGEN. All rights reserved.

Top Networks

ID	Associated Network Functions	Score
1	Cancer, Organismal Injury and Abnormalities, Reproductive System Disease	39
2	Cancer, Organismal Injury and Abnormalities, Reproductive System Disease	37
3	Cell Morphology, Cellular Compromise, Carbohydrate Metabolism	33
4	Cancer, Cellular Assembly and Organization, Gastrointestinal Disease	31
5	Cancer, Dermatological Diseases and Conditions, Hematological Disease	29

Top Tox Lists

Name	p-value	Ratio
RAR Activation	1,33E-03	16/173 (0,092)
Cardiac Hypertrophy	1,89E-03	27/375 (0,072)
PPARα/RXRα Activation	2,77E-03	15/169 (0,089)
Renal Necrosis/Cell Death	2,82E-03	31/463 (0,067)
Cardiac Necrosis/Cell Death	7,77E-03	19/262 (0,073)

(c) 2000-2014 QIAGEN. All rights reserved.

INGENUITY® SYSTEMS

Top My Lists

Name	p-value	Ratio

Top My Pathways

Name	p-value	Ratio

Top Molecules

This analysis has no expression values.

(c) 2000-2014 QIAGEN. All rights reserved.

INGENUITY® SYSTEMS
